# Seed and Foliar Application of Amino Acids Improve Variables of Nitrogen Metabolism and Productivity in Soybean Crop

**DOI:** 10.3389/fpls.2018.00396

**Published:** 2018-03-28

**Authors:** Walquíria F. Teixeira, Evandro B. Fagan, Luis H. Soares, Jérssica N. Soares, Klaus Reichardt, Durval D. Neto

**Affiliations:** ^1^Department of Crop Science, Luiz de Queiroz College of Agriculture, University of São Paulo, Piracicaba, Brazil; ^2^Department of Agronomy, Centro Universitário de Patos de Minas, Patos de Minas, Brazil

**Keywords:** glutamate, cysteine, phenylalanine, glycine, nitrogen

## Abstract

The application of amino acids in crops has been a common practice in recent years, although most of the time they are associated with products based on algae extracts or on fermented animal or vegetable wastes. However, little is known about the isolated effect of amino acids on the development of crops. Therefore, the objective of this research was to evaluate the effect of the application of isolated amino acids on the in some steps of the soybean nitrogen metabolism and on productivity. Experiments were carried out in a greenhouse and in the field with the application of the amino acids glutamate (Glu), phenylalanine (Phe), cysteine (Cys) and glycine (Gly) and as a set (Glu+Phe+Cys+Gly), as seed treatment (ST), as foliar application (FA) and both (ST+FA), at the V_4_ growth stage. Evaluations consisted of nitrate reductase and urease activities, nitrate, ureide, total amino acids and total nitrogen content in leaves, and productivity. The application of Glu to leaves, Cys as ST and a mixture of Glu+Cys+Phe+Gly as ST+FA in the greenhouse experiment increased the total amino acids content. In the field experiment all treatments increased the amino acid content in leaves. At the V_6_ stage in the field experiment, all modes of Gly application, Glu as ST and FA, Cys and Phe as ST+FA and Glu+Cys+Phe+Gly as FA increased the nitrate content in leaves. In the greenhouse, application of Cys and Phe as ST increased the production of soybean plants by at least 21%. The isolated application of Cys, Phe, Gly, Glu and the set of these amino acids as ST increased the productivity of soybean plants in the field experiment by at least 22%.

## Introduction

Nitrogen is an essential element for the development of plants and can be found in the soil in large quantities and in different chemical forms, such as inorganic ions as nitrate (NO_3_^-^) and ammonium (NH_4_^+^), or also complexed in organic molecules as proteins and amino acids. The main form of nitrogen uptake by the roots is via NO_3_^-^ and NH_4_^+^, or in the N_2_ form from the atmosphere by means of fixing bacteria (Jamtgard et al., 2010; Marschner, 2012).

However, although the inorganic form is the main route of nitrogen absorption by plants, several studies have been intensified in order to show the importance of organic forms of nitrogen for the roots. These studies show that plants such as wheat (Owen and Jones, 2001; Gioseffi et al., 2012), tomato (Ge et al., 2009) and boreal forest species (Persson and Nasholm, 2001) can absorb nitrogen in organic form, especially in simple forms such as amino acids.

The uptake of amino acids by plants is more advantageous energetically, when compared to the absorption of NO_3_^-^; NH_4_^+^ or biological fixation, because the plant does not need energy to assimilate the absorbed nitrogen and later incorporate it into amino acids (Jones and Kielland, 2002). However, the capacity of the amino acids to be absorbed by the roots is closely linked to their availability in the rhizosphere and to the activity of amino acid transporters in cell membranes in contact with the soil solution (Jamtgard et al., 2010).

Amino acids can play different roles in plants, such as stress-reducing agents, nitrogen source and hormone precursors (Zhao, 2010; Maeda and Dudareva, 2012). Another important fact is the role of amino acids as a signaling factor of different physiological processes in plants. In *Arabidopsis thaliana*, glutamate receptors (GRLs) have been identified, which are also capable of binding to other amino acids (Vincill et al., 2012; Forde and Roberts, 2014). These receptors, when activated by amino acids, are capable of triggering a series of physiological processes such as the regulation of nitrogen uptake (Miller et al., 2007), root development (Walch-Liu and Forde, 2007; Weiland et al., 2015) and antioxidant metabolism (Hildebrandt et al., 2015; Weiland et al., 2015). The better root growth favored by the addition of amino acids can enhance the biologic nitrogen fixation, which leads to a greater production of ureides. Furthermore, with the larger root surface the nutrient uptake can also be increased, as for example, the nitrate. The nitrogen metabolism can also be altered due to the signaling role promoted by the amino acids.

Recently, Santi et al. (2017) showed that maize plants grown in a diluted nutrient solution containing a mixture of free amino acids presented changes, and included several transcripts encoding transcription factors, among them those related to cellular organization, stress, transport and metabolism and hormonal signaling.

Some studies have shown the efficiency of amino acid uptake by plants (Persson et al., 2003; Gioseffi et al., 2012), and that the application of amino acids via seed treatment may have an effect on a good development of the plant, since these molecules can trigger physiological processes favorable to development. Other studies show a positive effect of foliar application of amino acid mixtures on plants, such as increased productivity in *Solanum lycopersicum* (Koukounaras et al., 2013) and higher accumulation of dry matter mass, chlorophylls, carbohydrates and polysaccharides in *Vicia faba* (Sadak et al., 2014).

Although the effect of amino acids application has been documented in recent years, few works are related to the soybean crop. In addition, most of the studies are conducted using a group of amino acids in only one application time, not allowing the characterization of the effects of isolated amino acids and of the modes of their application. We have recently shown (Teixeira et al., 2017) that the amino acids used in this work have different effects on the oxidative metabolism of soybean as a function of the application form (seed treatment, foliar application or foliar seed treatment). Thus, this study is based on the hypothesis that the application of amino acids can modulate some steps involved in the nitrogen metabolism, which can lead to increasing the productivity of the soybean crop.

Therefore, the objective of the present work was to evaluate the effect of the application of glutamate, cysteine, phenylalanine, and glycine applied isolated or combined in seed treatment, foliar application or foliar + seed treatment, in some steps of the nitrogen metabolism (nitrate reductase and urease activity and nitrate, ureide, total amino acids and total nitrogen content in leaves), and the productivity of the soybean crop.

## Materials and Methods

### Experiments

Two experiments were conducted, one in a greenhouse and one in the field. The treatments used in both experiments were applications of solutions of glutamate (Glu), phenylalanine (Phe), glycine (Gly), cysteine (Cys) and all amino acids in association (Glu+Phe+Cys+Gly), on seeds (seed treatment, ST), on leaves at vegetative stage V_4_ (foliar application, FA) and both (ST+FA). A control treatment, in which only distilled water was applied on seeds and leaves, was also included in the experimental design (**Table 1**).

**Table 1:** Treatments with different amino acids applied only on seed (ST), mg kg^-1^ (seeds), foliar application (FA) at V_4_ (mg ha^-1^) only, and both ST+FA.

Amino acids^1^	Moment of application			
	
	ST	FA	Both ST+FA	
Control	0	0	0	0
Glutamate (Glu)	12	123	12	123
Cysteine (Cys)	12	123	12	123
Phenylalanine (Phe)	3	30	3	30
Glycine (Gly)	9	92	9	92
Glu+Cys+Phe+Gly	12 + 12 + 3 + 9	123 + 123 + 30 + 92	12 + 12 + 3 + 9	123 + 123 + 30 + 92


*^*1*^Values with higher weights within principal component (PC).*

#### Greenhouse

This experiment was conducted in a greenhouse located in the Plant Production Department of the “Luiz de Queiroz” School of Agriculture (Esalq/USP), Piracicaba municipality, SP (22°41′S, 47°38′W and 546 m altitude).

Pots with 11 dm^3^ capacity, containing washed sand as substrate were used to plant the soybean (*Glycine max* L. Merrill) cultivated variety NS 7901 RR. Ten seeds were sown per pot and, after emergence, plants were thinned to three plants per pot. The experiment was conducted in completely randomized block design with 12 blocks, and all the treatments distributed in each block.

During the conduction of the experiment pots were irrigated daily according to the water requirement (400 mL per pot). A weekly application of a nutrient solution was applied, as proposed by Johnson et al. (1957).

Foliar application of amino acids was performed taking into consideration the amount of plants in each treatment. The rate of the amino acids was that recommended per ha, and also the recommended population for cultivated variety used in this study of 250,000 plants ha^-1^. Based on these recommendations, the amount of the amino acid to be applied on 36 plants per treatment was calculated.

#### Field

The experiment was conducted in an experimental area of the Agro-technical Afonso Queiroz School, Campus II of Unipam (University Center of Patos de Minas), municipality of Patos de Minas (MG) (18° 34′ S, 46° 31′ W, altitude 815 m). The soil of the site is classified as an Oxisol (Soil Survey Staff, 2014) and the area presents a tropical climate of altitude (Cwa, according to Köppen), with annual average precipitation around 1,400 mm (Souza et al., 2005).

Based on soil analysis, 36 kg ha^-1^ of N, 54.6 kg ha^-1^ of P, 37.5 kg ha^-1^ of K, 10.5 kg ha^-1^ of Ca, 19.1 kg ha^-1^ S, 0.9 kg ha^-1^ B and 0.9 kg ha^-1^ of Zn were applied before sowing. For weed control the herbicide glyphosate was applied at 17 and 32 days after sowing (DAS) [650 g a.i. U^-1^ at a rate of 2.2 kg b.w. ha^-1^]. For insect control, methomyl [215 g a.i. ha^-1^ at a rate of 1.5 U b.w. ha^-1^] was used at 80 and 106 DAS, and methamidophos [600 g a.i. L^-1^ at a rate of 1.0 L p.c. ha^-1^] at 90 DAS. Disease control was promoted by application of pyraclostrobin and epoxiconazole [133 g a.i. ha^-1^, 50 g a.i. ha^-1^, respectively, at a rate of 0.6 L p.c. ha^-1^], and also carbendazim [663 g a.i. U^-1^ at a rate of 0.6 U b.w. ha^-1^] at 90 and 106 DAS.

The experiment was conducted in completely randomized block design with eight blocks, and all the treatments distributed in each block, using the same greenhouse experiment treatments (**Table 1**).

Soybean of the cultivated variety NS 7901 RR was seeded, with a population at harvest of 250,000 plants ha^-1^. Each plot was composed of four lines 7 m long by 0.45 m between rows, defining plots of 12.6 m^2^. The sampling area of each plot was constituted of the two central lines, discarding 0.5 m at each end of the plot.

Foliar applications were performed at the V_4_ stage (four nodes on the main stem) stage with a CO_2_ propellant sprayer. The bar used contained four fan-type nozzles, being 2.25 m long and with a pressure of 2 bar. For all applications, a volume of 200 L ha^-1^ solution was used.

### Assessments

#### Nitrate Reductase and Urease Activity

For the determination of nitrate reductase and urease activity, completely expanded leaves were collected from the middle third of five plants in each replicate.

Urease was evaluated when the plants were at stage V_6_ (six nodes on the main stem). Extraction of the material for the determination of the urease activity was performed in fresh material according to the methodology adapted from Hogan et al. (1983). The determination of N-NH_4_^+^ was performed according to the methodology proposed by McCullough (1967). The activity of the enzyme was determined by the amount of NH_4_^+^ produced, and the values obtained were compared with a standard curve of ammonium chloride (NH_4_Cl), and the results were expressed in μmol [NH_4_^+^] h^-1^ g^-1^ [fresh matter mass].

The determination of nitrate reductase was performed only in the field experiment, at stages V_6_. This analysis was performed according to the method proposed by Mulder et al. (1959), with results expressed in μg [N-NO_2_] g^-1^ [fresh matter mass] h^-1^.

#### Nitrate, Ureide, Total Amino Acids and Total Nitrogen Content in Leaves

All these evaluations were carried out at stage V_6_. Completely expanded leaves were collected from the middle third of five plants of each replicate. This material was dried in an oven with forced air circulation at a temperature of 65°C and later crushed with the aid of an IKA glass mill. For the determination of nitrate (NO_3_^-^), total amino acids (Aa) and ureide, 200 mg of plant material was transferred to falcon tube and 10 mL of Milliq water were added. After this procedure, the material was incubated at 45°C for 1 h and then centrifuged at 10,000 rpm for 15 min. At the end, the supernatant was separated and stored in a freezer for further evaluation.

The quantification of NO_3_^-^ was performed by the method proposed by Cataldo et al. (1975). For the determination of total amino acids, the protocol of Yemm and Cocking (1955) with adaptations described in Herridge (1984) was used. The ureides were determined based on the method proposed by Young and Conway (1942).

Finally, quantification of the total nitrogen was carried out, for which the dry and ground leaves were digested in a solution with sulfuric acid and later, the nitrogen content was determined by the Kjeldahl method (Silva, 1999).

#### Productivity

In the greenhouse experiment, plants were harvested manually, considering three plants per replicate. The grains harvested from each plant were weighed on a digital scale with an accuracy of 0.01 g. The water content of the grain was determined and the productivity was calculated with the water content corrected to 13% (0.13 g g^-1^). In the field experiment, the plants were harvested manually considering the two central rows. At the end the material was weighed and determinations were made as described in the greenhouse experiment.

### Statistical Analysis

Data of the two experiments were evaluated for normality and homogeneity using the Shapiro–Wilk and Levene tests, respectively, both at the 5% significance level.

We performed statistical analysis as one-way ANOVA. Therefore, the variance analysis was performed and, when significant, the Duncan test was applied at the 5% level of significance.

For the field experiment, the multivariate analysis was performed through Principal Component Analysis. All analyzes were performed using the statistical software SAS 9.3 (SAS Institute, 2011).

## Results

### Greenhouse Experiment

**FIGURE 1:**
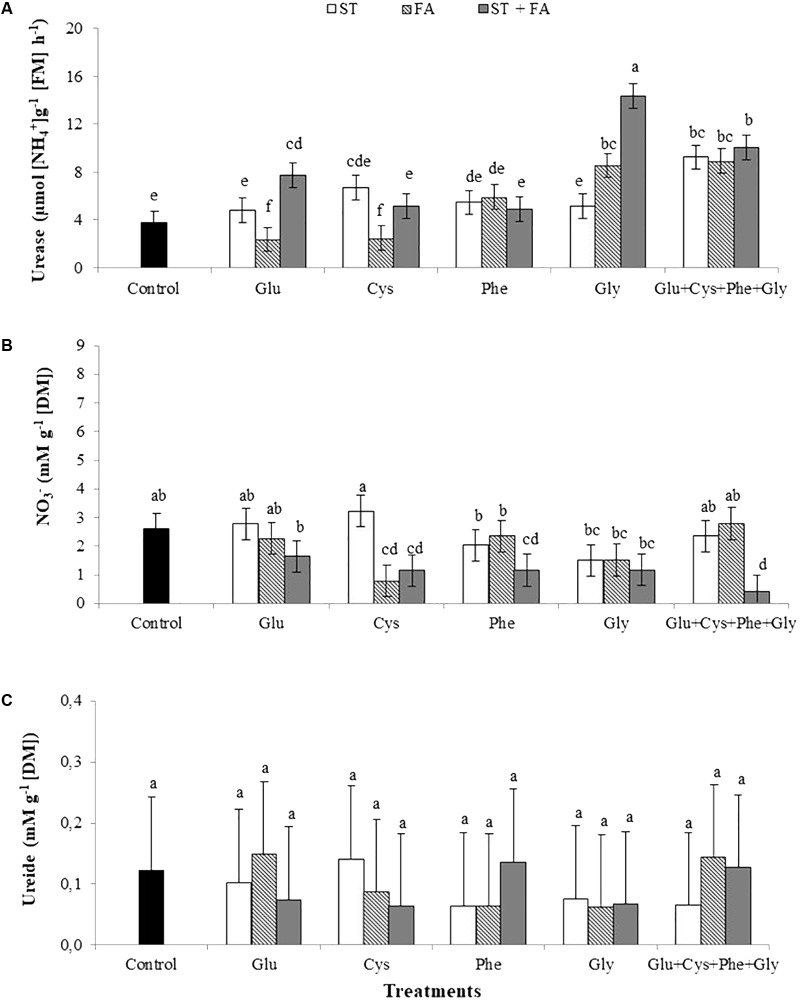
Effects of use of glutamate (Glu), cysteine (Cys), phenylalanine (Phe), glycine (Gly) and all these amino acids in association (Glu+Cys+Phe+Gly), as a function of seed treatment (ST) and/or foliar application (FA), on the soybean leaf urease activity **(A)**, nitrate (NO_3_^-^, **B**) and ureide **(C)** at V_6_ stage. Greenhouse experiment. Season 2015/2016. Means followed by the same letters do not differ significantly from each other, using the Duncan test at 5% significance.

The application of glycine as ST+FA and FA only increased urease activity at the V_6_ growth stage (**Figure 1A**). The use of the amino acid set in all modes of application also increased the activity of this enzyme.

At the V_6_ stage the nitrate content in leaves was reduced with the application of cysteine as FA and as ST+FA, phenylalanine as ST+FA and use of the amino acids set applied as ST+FA (**Figure 1B**).

No difference in the content of ureide in leaves was observed for the application of amino acids (**Figure 1C**).

**FIGURE 2:**
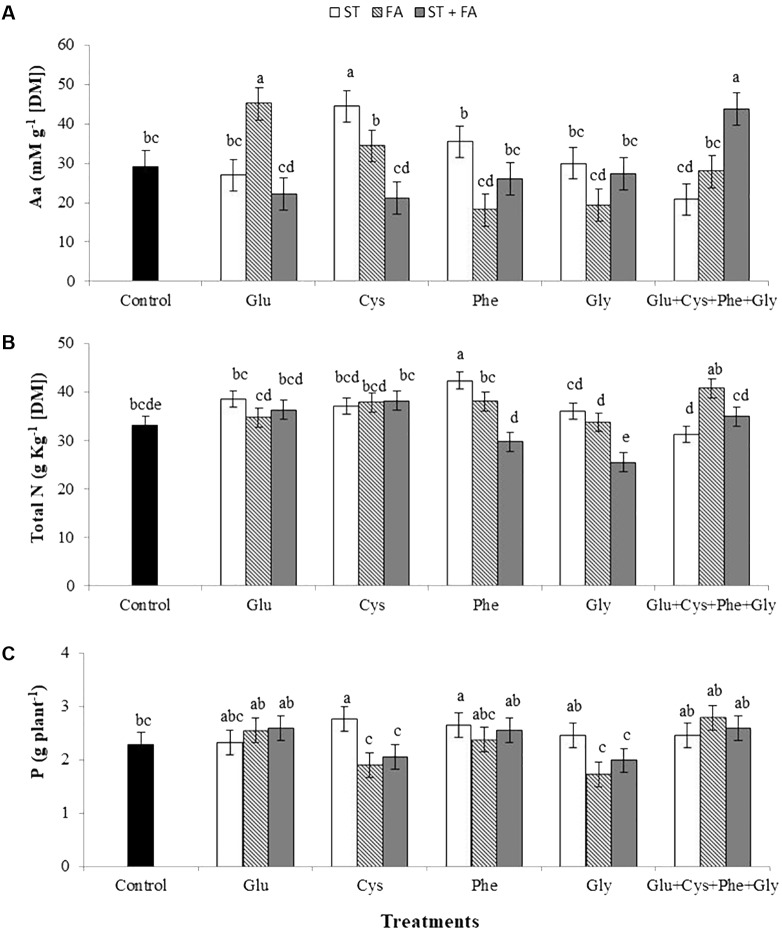
Effects of use of glutamate (Glu), cysteine (Cys), phenylalanine (Phe), glycine (Gly) and all these amino acids in association (Glu+Cys+Phe+Gly), as function of seed treatment (ST) and/or foliar application (FA), on the total amino acid (Aa, **A**) and total nitrogen (Total N, **B**) at the V_6_ stage and soybean productivity (P, **C**). Greenhouse experiment. Season 2015/2016. Means followed by the same letters do not differ significantly from each other, using the Duncan test at 5% significance.

However, the use of cysteine as ST, glutamate as FA and of the amino acid set as ST+FA increased the total amino acid content in leaves at the V_6_ stage (**Figure 2A**). Moreover, only phenylalanine applied as ST increased the total nitrogen content (**Figure 2B**).

The use of cysteine and phenylalanine in ST increased the productivity by 21 and 16%, respectively, while the other treatments did not differ from the control (**Figure 2C**).

### Field Experiment

**FIGURE 3:**
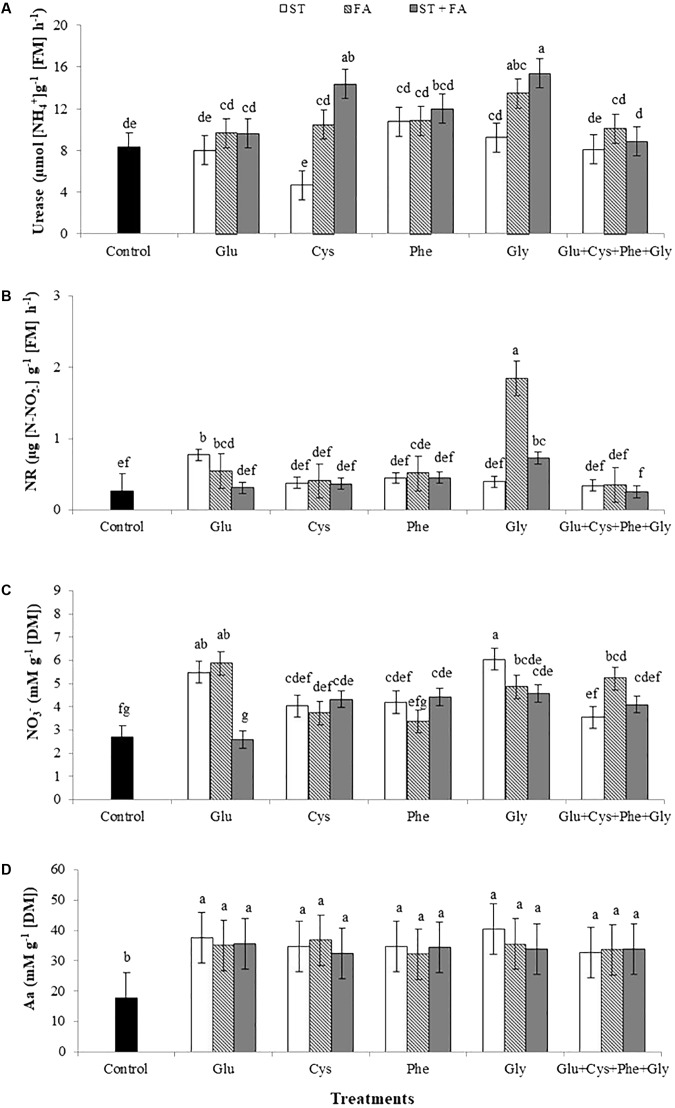
Effects of use of glutamate (Glu), cysteine (Cys), phenylalanine (Phe), glycine (Gly) and all these amino acids in association (Glu+Cys+Phe+Gly), as a function of seed treatment (ST) and/or foliar application (FA), on the soybean leaf urease activity **(A)**, nitrate reductase activity (NR, **B**), nitrate (NO_3_^-^, **C**) and total amino acid (Aa, **D**) at the V_6_ stage. Field experiment. Season 2014/2015. Means followed by the same letters do not differ significantly from each other, using the Duncan test at 5% significance.

The comparison of all treatments leads to the observation that the use of glycine and cysteine at ST+FA and glycine only in FA increased the urease activity (**Figure 3A**).

The application of glycine as FA and glutamate as ST and FA increased the nitrate reductase activity evaluated at V_6_ stage (**Figure 3B**).

Under field conditions the plant response to nitrate uptake differed from the greenhouse experiment, since the application of several treatments with amino acids increased the nitrate content in leaves at V_6_ stage. The application of glutamate and glycine as ST, glutamate, glycine and the set of amino acids as FA, and cysteine, phenylalanine and glycine as ST+FA increased the nitrate content in leaves (**Figure 3C**).

**FIGURE 4:**
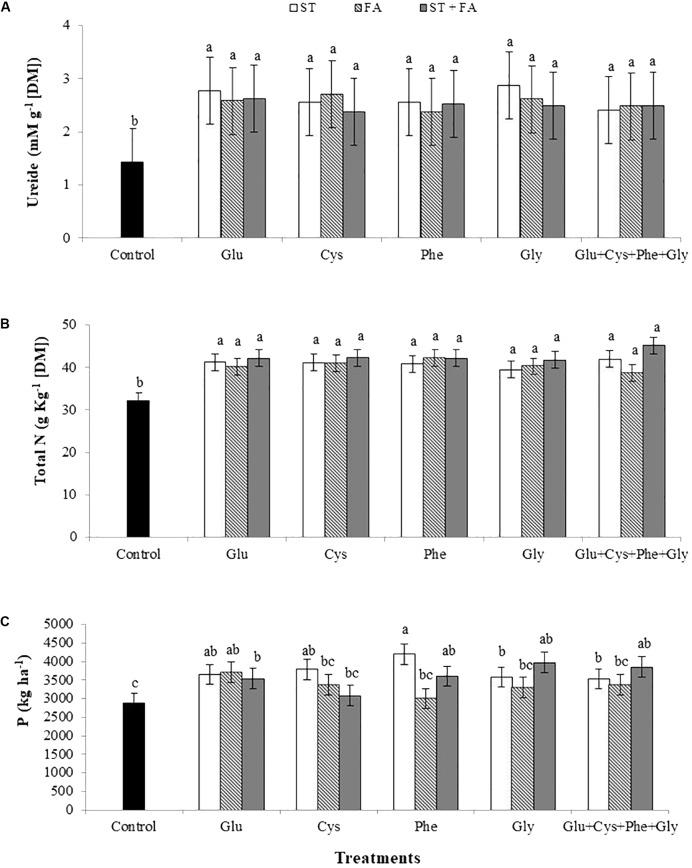
Effects of use of glutamate (Glu), cysteine (Cys), phenylalanine (Phe), glycine (Gly) and all these amino acids in association (Glu+Cys+Phe+Gly), as a function of seed treatment (ST) and/or foliar application (FA), on the ureide **(A)**, total nitrogen (Total N, **B**) at V_6_ stage and productivity (P, **C**). Field experiment. Season 2014/2015. Means followed by the same letters do not differ significantly from each other, using the Duncan test at 5% significance.

For all treatments the total amino acids content increased in relation to the control (**Figure 3D**), the same occurred for the ureide content (**Figure 4A**) and total nitrogen in leaves (**Figure 4B**).

The most expressive increase in productivity was obtained by the application of phenylalanine as ST, 46% higher than the control. The other treatments also increased productivity, except cysteine, phenylalanine, glycine and the association of all amino acids at FA and cysteine at ST+FA (**Figure 4C**).

**FIGURE 5:**
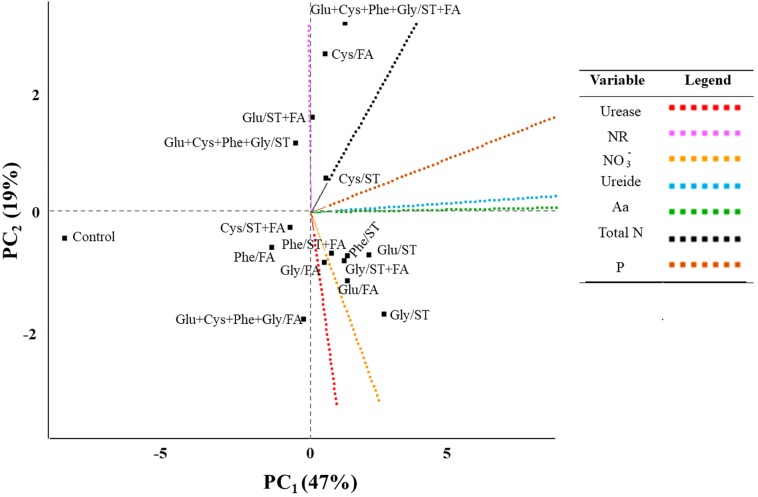
Byplot obtained from the analysis of main components of the results of use of glutamate (Glu), cysteine (Cys), phenylalanine (Phe), glycine (Gly) and all these amino acids in association (Glu+Cys+Phe+Gly), as a function of seed treatment (ST) and/or foliar application (FA), on the soybean leaf activity of urease, NR_1_, nitrate reductase; NO_3_^-^, nitrate; ureide; Aa, total amino acids; and Total N, total nitrogen at V_6_ stage; P, productivity. Field experiment. Season 2014/2015.

From the analysis of main components (PC) two variables were obtained with the greatest representativeness (**Figure 5**), ureide and total amino acids as PC_1_ and nitrate reductase as PC_2_ (**Table 2**). These variables showed greater weight in the variance. The productivity correlated with the nitrogen content and ureide in leaves. According to the analysis the use of cysteine in the treatment of seeds provided higher productivity. The control treatment presented low values for all analyzed variables.

**Table 2:** Eigen values, percentage of variance, cumulative variance, eigen vectors for different principal components.

Principal component (PC)	Eigen values	Percentage of variance	Cumulative variance	Eigen vectors		
				
				Variable	PC_1_	PC_2_
1	3.26	0.47	0.47	U	0.11	-0.26
2	1.29	0.19	0.66	NR	-0.03	0.85^1^
4	1.05	0.15	0.81	NO_3_^-^	0.62	-0.56
5	0.66	0.09	0.90	Ureide	0.96^1^	0.02
6	0.53	0.08	0.98	Aa	0.96^1^	0.00
7	0.19	0.01	0.99	NTotal	0.74	0.43
8	0.00	0.01	1.00	P	0.68	0.08


*^*1*^Values with higher weights within principal component (PC).*

**FIGURE 6:**
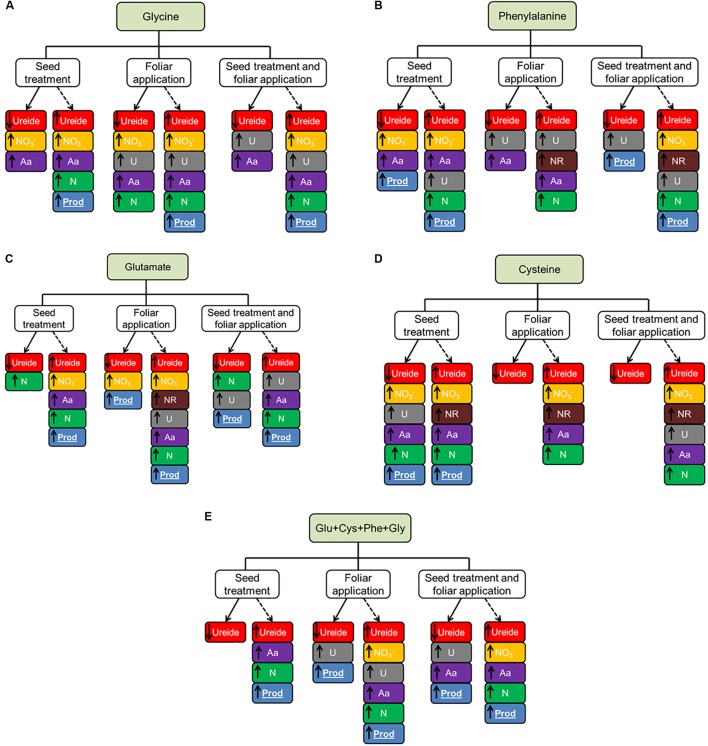
Summary of the effect of glycine (Gly, **A**), phenylalanine (Phe, **B**), glutamate (Glu, **C**), cysteine (Cys, **D**) and set of amino acids (Glu+Cys+Phe+Gly) **(E)**, applied in the seed treatment, foliar aplication and both, in soybean crop in greenhouse experiment (continuous line) and field experiment (dotted line).

A synthesis of the results obtained by applying the amino acids is summarized in **Figure 6**. From these results we can observe some common responses that were observed in the two experiments. The use of leaf glycine and as ST+FA provided the increased urease activity in both experiments.

Phenylalanine in seed treatment increased the overall nitrogen content and productivity. The application of cysteine only showed a common response in the two experiments when carried out as seed treatment, where it provided the increase of total amino acids and productivity.

The use of glutamate in the leaves provided the increase of the amino acid content in the plants. This same amino acid applied as ST+FA increased the urease activity. Finally, the set of amino acids applied as ST+FA increased the total amino acids content and the ST or FA application increased the urea activity in both experiments.

## Discussion

Although amino acid application is a recurring practice in world agriculture, most of the research was carried out for horticultural crops. In addition, most of the work was performed with biostimulants containing a mixture of amino acids (Colla et al., 2015). In this sense, this study reports how pure amino acids, applied in isolation or together modulate biochemical variables involved in the nitrogen metabolism of the soybean crop.

As shown in the results, in general the amino acid application increases variables related to nitrogen metabolism in soybean, such as nitrate, amino acids and total nitrogen content. However, Teixeira (2017) has already shown that the amount of N provided by the application of amino acids via ST, FA, or ST+FA represents less than 1% of the amino acid content already present in the leaves. Therefore, in this work it is believed that the use of amino acids applied to seeds and on leaves is not a source of nitrogen for the plant, and yes, they are molecules that can act as signals in different metabolic processes, thus inducing greater assimilation of nitrogen by plants (Santi et al., 2017).

Some studies indicate that there are glutamate receptors (GLRs) in plants (Price et al., 2012; Forde and Roberts, 2014). These studies reveal that GRLs can be activated by amino acids other than glutamate, including L-glutamate, L-serine, L-alanine, methionine, tryptophan, phenylalanine, leucine, asparagine, threonine, cysteine, glycine, tyrosine and peptides such as glutathione (GSH) (Vincill et al., 2012; Forde and Roberts, 2014).

Glutamate receptors are able to mediate a number of plant responses such as changes in root architecture, plant stress signaling, carbon metabolism, stomatal movements, photosynthesis and plant immunity (Weiland et al., 2015). Moreover, through the signaling provided by the activation of the GLRs, it is possible to change the nitrogen metabolism as well as the C/N balance in plants (Kang and Turano, 2003; Price et al., 2012).

In our work, the amino acids altered some variables of nitrogen metabolism. The application of glutamate in FA induced higher total amino acid accumulation (**Figures 2A**, **3D**) and ST+FA of this amino acid increased the urease activity at the V_6_ stage under greenhouse and field conditions (**Figures 1A**, **3A**). The role of glutamate in signaling and activation of GRLs lead to a greater root development, which provided greater nitrogen uptake. According to Walch-Liu and Forde (2007), glutamate causes inhibition of primary root growth in *Arabidopsis* and the consequent increases development of secondary roots, increasing the nutrient uptake capacity of plants.

In addition, Santi et al. (2017) observed in their work that the application of amino acids increased the transcription of genes involved in the transport of nitrate, ammonium, phosphate, magnesium, and iron. Therefore, the effect observed in our work may be related to the fact that the amino acids provided an increase in nitrogen assimilation, due to the increase in nitrate reductase activity (**Figure 3B**). Among the factors that regulate nitrate reductase activity, nitrate availability and plant metabolic status are important factors (Kaiser and Huber, 2001). It has already been demonstrated by us that the use of amino acids reduce plant stress (Teixeira et al., 2017). Therefore, the reduction in the stress level associated with higher nitrate availability provided the increase in nitrate reductase activity (**Figure 3B**). Nitrate reductase depends directly on the energy coming from the photosynthesis [NAD(P)H], in this way, the good physiological status of the plant affects the increase of enzyme activity (Marschner, 2012). Therefore, possibly the amino acids allowed the plants to direct greater energy for the assimilation of nitrogen via nitrate reductase. In addition, the role of amino acids as signaling elements may have led to increased nitrate reductase activity, since the activation of amino acids mediated GLRs may have increased the activity of enzymes linked to nitrogen metabolism (Kang and Turano, 2003; Price et al., 2012).

The increment of the nitrate reductase activity increased the nitrite content in the leaf. The nitrite may undergo action of the enzyme nitrite reductase that produces ammonium, which in turn can be incorporated into amino acids (Taiz and Zeiger, 2013). This explains the increase in amino acid content as a result of the application of glutamate (**Figure 3D**) to leaves.

Another characteristic of glutamate is that it is involved in several metabolic routes in plants, among them, the synthesis of other amino acids such as arginine, proline, aspartate, and glutamine (Taiz and Zeiger, 2013). These amino acids may affect the content of several compounds in the plant, as evidenced by Amin et al. (2011). These authors demonstrated that glutamine application in *Allium cepa* plants increased total amino acids, soluble sugars and phenolic compounds.

The use of cysteine as ST, glutamate as FA and set of amino acids as ST+FA increased the amino acid content in the leaves, in both experiments. These results do not corroborate with other studies that show that the application of 1 mM of asparagine, glutamine, or glutamate leads to a reduction of other forms of nitrogen in the plant, mainly nitrate, ammonium, and total nitrogen (Vidmar et al., 2000; Miller et al., 2007). Nagao et al. (2005) carried out an experiment with the application of proline and inosine to plants of *Lolium multiflorum* and observed that these amino acids did not alter the total nitrogen content in leaves. Another study shows that the application of 1 mM of glutamine on seeds of *Hordeum vulgare* did not alter the nitrate content in plants; however, it reduced the nitrate reductase activity (Fan et al., 2006).

The use of glycine in FA and ST+FA elevated urease activity in both experiments (**Figures 1A**, **3A**). This enzyme performs the conversion of urea into ammonia, which is rapidly converted to ammonium. The production of this urea can occur naturally in plants, from several compounds, among them arginine, during the production of ornithine, and from allantoic acid, which is a type of ureide, coming from biological fixation (Marschner, 2012). The application of glycine provided the increase of ureides in field conditions. This compound in addition to being incorporated into amino acids may have taken an alternative route, with the consequent production of urea and increased urease activity.

The ureide content was increased with the application of all treatments in the field experiment conditions (**Figure 4A**), which allows to infer that there was greater biological fixation by the plants. This effect may have been provided due to the larger root area as a result of amino acids application, which means a greater contact area for nodulation. Some amino acids, such as glutamate, cause inhibition of primary root growth and the consequent increased development of secondary roots (Walch-Liu and Forde, 2007). On the other hand, Horváth et al. (2015) demonstrate that the capacity of nodulation in Fabaceae (*Medicago truncatula*) is directly related to the presence of cysteine residues, which act in the formation of nodules. In the greenhouse experiment, no effect of the treatments on the ureide content was observed due to the experimental conditions (sand experiment), which did not favor the nodulation of the plants.

In addition, it is speculated that the effects on biological nitrogen fixation may be related to the signaling triggered by amino acids. In the work of Santi et al. (2017), it was observed that the amino acids altered the transcription of genes involved in cytokinin homeostasis in maize (*Zea mays* L.) plants, which probably resulted in a higher free cytokinin content in the plants. It is known that cytokinins are important for the biological fixation of nitrogen, regulating mainly the organogenesis of the root cortex during nodulation (Ryu et al., 2012). Therefore, these treatments possibly potentiated the biological fixation of nitrogen. However, there are still no studies that show how the application of amino acids interacts to regulate the biological fixation of nitrogen in soybean.

All these positive characteristics of the use of amino acids had repercussions in the greater productivity of the plants. The use of phenylalanine and cysteine in the seed treatment in both experiments (**Figures 2C**, **4C**), glutamate, glycine and the set of amino acids applied in ST and in both modes in the field experiment (**Figure 4C**) led to higher productivity.

It has been reported in the literature that the use of the amino acid pool may be more effective in productivity. In an experiment carried out with *V. faba* the use of a mixture of amino acids from a commercial product (aspartic acid, serine, glutamate, proline, lysine, methionine, isoleucine, leucine, tyrosine, phenylalanine, histidine and arginine at a dose of 1500 mg L^-1^), applied to leaf, provided an increase in the dry matter mass of the plants, besides increasing the carbohydrate content, polysaccharides and plant productivity (Sadak et al., 2014).

On the other hand, Soares et al. (2016) found that the application of an amino acid mixture to seeds (glutamate, cysteine, glycine, arginine, and methionine at the doses of 31, 30, 34, 42, and 37 mg kg^-1^ of seeds, respectively) resulted in increased productivity in soybean plants grown in the field.

The fact that the greatest increase in productivity was obtained when amino acids were applied in seeds, is probably related to their role in modulating root architecture. Forde (2014) shows that several amino acids, among those used in this work, cause changes in the formation of the main and lateral roots of *A. thaliana*. Teixeira (2017) also showed that the amino acids used in this work alter, among other parameters, root volume and number of lateral roots of soybean plants. Therefore, when amino acids are used in seed treatment, if changes occur in the architecture of the roots, the plants that allow more efficient use of the water and soil nutrients can increase productivity.

Therefore, these results reinforce the idea that the effect provided by the application of amino acids is not related to their direct use in plant metabolism, but rather due to signaling the effects that they perform, as previously written, since the amount applied is very low in relation to the content naturally present in the leaves.

## Conclusion

The present study demonstrated biostimulant action of amino acids in soybean plants. It has been shown that the use of amino acids in seed treatment is more efficient in relation to productivity.

The application of cysteine, phenylalanine, glycine, glutamate and all these amino acids (Glu+Phe+Cys+Gly) together can increase nitrate, amino acids and total nitrogen contents in soybean leaves, possibly due to the signaling action of these amino acids.

Future research should focus the evaluation of the mechanisms of how these amino acids can affect the genetic transcription of different parameters, including nutrient transporters, hormone production and antioxidant metabolism. In this way, it will be possible to obtain the best understanding about the role of these amino acids as biostimulants in soybean plants.

## Author Contributions

WT, EF, and DN designed the experiments. WT performed the experiments, including plant analysis and wrote the manuscript. JS assisted in the performance of most of the experiments and plant analysis. LS assisted in the performance of most of the experiments and preparation of the manuscript. KR performed the correction and translation of the manuscript. All the authors discussed the results and revised the manuscript.

## Conflict of Interest Statement

The authors declare that the research was conducted in the absence of any commercial or financial relationships that could be construed as a potential conflict of interest.
